# Co-infection of SARS-CoV-2 and influenza virus causes more severe and prolonged pneumonia in hamsters

**DOI:** 10.1038/s41598-021-00809-2

**Published:** 2021-10-28

**Authors:** Takaaki Kinoshita, Kenichi Watanabe, Yasuteru Sakurai, Kodai Nishi, Rokusuke Yoshikawa, Jiro Yasuda

**Affiliations:** 1 Department of Emerging Infectious Diseases, Institute of Tropical Medicine (NEKKEN), Nagasaki University, 1-12-4 Sakamoto, Nagasaki, Nagasaki 852-8523 Japan; 2 National Research Center for the Control and Prevention of Infectious Diseases (CCPID), Nagasaki University, Nagasaki, Japan; 3 Research Center for Global Agromedicine, Obihiro University of Agriculture and Veterinary Medicine, Obihiro, Japan; 4 Department of Radioisotope Medicine, Atomic Bomb Disease Institute, Nagasaki University, Nagasaki, Japan; 5 Graduate School of Biomedical Science, Nagasaki University, Nagasaki, Japan

**Keywords:** Infectious diseases, Virology

## Abstract

Coronavirus disease 2019 (COVID-19) caused by severe acute respiratory syndrome coronavirus 2 (SARS-CoV-2) is currently a serious public health concern worldwide. Notably, co-infection with other pathogens may worsen the severity of COVID-19 symptoms and increase fatality. Here, we show that co-infection with influenza A virus (IAV) causes more severe body weight loss and more severe and prolonged pneumonia in SARS-CoV-2-infected hamsters. Each virus can efficiently spread in the lungs without interference by the other. However, in immunohistochemical analyses, SARS-CoV-2 and IAV were not detected at the same sites in the respiratory organs of co-infected hamsters, suggesting that either the two viruses may have different cell tropisms in vivo or each virus may inhibit the infection and/or growth of the other within a cell or adjacent areas in the organs. Furthermore, a significant increase in IL-6 was detected in the sera of hamsters co-infected with SARS-CoV-2 and IAV at 7 and 10 days post-infection, suggesting that IL-6 may be involved in the increased severity of pneumonia. Our results strongly suggest that IAV co-infection with SARS-CoV-2 can have serious health risks and increased caution should be applied in such cases.

## Introduction

In December 2019, the first cases of coronavirus disease 2019 (COVID-19), caused by severe acute respiratory syndrome coronavirus 2 (SARS-CoV-2), were reported in Wuhan, China. Since then, it has spread globally and the outbreak has been declared a pandemic. As of May 2021, the pandemic is still ongoing, with significant increase in cases each day.

The clinical spectrum of patients infected with SARS-CoV-2 is quite broad. Although the current case fatality is around 2%^1^, co-infection with other pathogens may worsen the COVID-19 symptoms and increase fatality. Among the pathogens that can establish a co-infection with SARS-CoV-2, influenza A virus (IAV) is one of the most likely candidates, because around one billion people are estimated to be infected with this virus every year, especially in the winter season. Both SARS-CoV-2 and IAV follow similar transmission routes and mainly cause respiratory diseases. Indeed, quite a few cases of co-infection by these viruses have been reported^2–6^. However, the number of cases is not high enough to conclude how co-infection by IAV affects the symptoms of COVID-19. As both the pathogens commonly affect the respiratory system, co-infection may enhance the pathogenic effects. Contrastingly, it is also possible that SARS-CoV-2 and IAV may inhibit each other’s infection and replication, since co-infecting viruses have been reported to interfere with the replication of the other^7^.

The hamster model has been established as an animal model for SARS-CoV-2 infection, since hamsters are susceptible to SARS-CoV-2 infection and show symptoms, including pneumonia, mimicking those of COVID-19 in humans^8,9^. Hamsters infected with a high dose of SARS-CoV-2 showed significant body weight loss by 7 days post infection with the progression of pneumonia and then recovered by day 14. Efficient viral replication was observed in the lung of hamsters, where the cellular receptor of SARS-CoV-2, angiotensin converting enzyme 2 (ACE2), is present^10^. Hamsters have also been used in studies on potential therapeutic antibodies and compounds against SARS-CoV-2^11–15^. They are also used as an animal model for human influenza virus infection^16^.

Therefore, in this study, we investigated the effects of IAV co-infection on the pathogenicity and the in vivo proliferation of SARS-CoV-2 using a hamster model.

## Results

### Body weight change by infection

To investigate if the pathogenesis of SARS-CoV-2 is altered by IAV co-infection, hamsters were infected with SARS-CoV-2, IAV, or both. In this study, we used a mouse-adapted influenza A/Puerto Rico/8/34 (H1N1) virus (PR8) and a clinical isolate of SARS-CoV-2, JPN/NGS/SC-1/2020, which was isolated from a fatal case.

**Figure 1:**
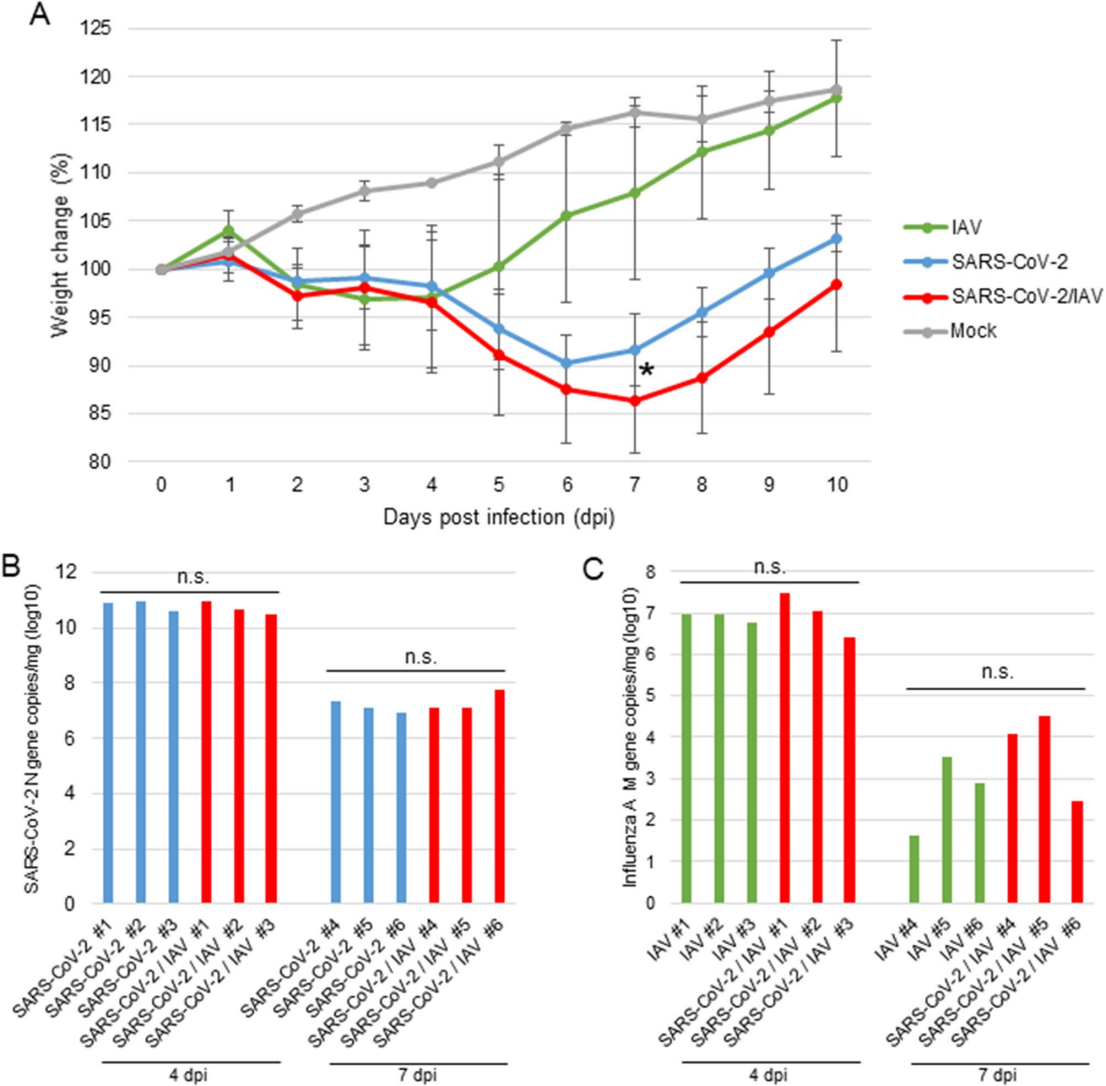
Effect of co-infection on body weight and virus replication. (**A**) Body weight changes in Syrian hamsters during infection. Hamsters (n = 6 per group) were intranasally inoculated with 1 × 10^5^ PFU of IAV (PR8), 3 × 10^5^ PFU of SARS-CoV-2, or a mixture of both viruses (co-infection). The hamsters of the mock-infected group were administered DMEM. Mean percentage increase or decrease in body weight at 0 dpi ± SD is shown at each consecutive dpi.* P* values were calculated using Tukey’s multiple-comparison test (*P < 0.05; between SARS-CoV-2 and SARS-CoV-2/IAV). (**B**) SARS-CoV-2 viral RNA was detected by RT-qPCR for SARS-CoV-2N gene in the lung homogenate at 4 and 7 dpi. N gene copies per lung weight (mg) were recorded at 4 and 7 dpi for each treatment. (**C**) IAV viral RNA was determined by RT-qPCR for IAV M gene in lung homogenate at 4 and 7 dpi. M gene copies per lung weight (mg) were recorded at 4 and 7 dpi for each treatment. *P* values were calculated using Student’s t-test. (n.s.: not significant).

In a group of hamsters infected with only IAV (PR8), the body weight slightly decreased until 3 days post-infection (dpi) and then gradually increased (Fig. 1A). At 10 dpi, the body weights were similar to those of mock-infected hamsters. By contrast, hamsters infected with only SARS-CoV-2 or both SARS-CoV-2 and IAV showed significant decrease in body weight until 6 or 7 dpi, respectively. Co-infected hamsters showed more significant body weight loss than those infected with only SARS-CoV-2 at 7 dpi. In addition, body weight recovery in co-infected hamsters was delayed by a day compared with that in hamsters infected with only SARS-CoV-2.

### Virus replication in the lungs

Next, we analyzed the virus replication in the lungs of infected hamsters at 4 and 7 dpi by real time RT-qPCR. SARS-CoV-2 viral RNA load in the lungs at 4 dpi was higher than that at 7 dpi, whereas there was no significant difference in the viral RNA levels in the lungs of SARS-CoV-2 single-infected and co-infected hamsters at both 4 and 7 dpi (Fig. 1B). Similarly, the level of IAV RNA was higher at 4 dpi, and both IAV single-infected and co-infected hamsters showed similar levels of IAV RNA at 4 and 7 dpi (Fig. 1C).

### X-ray microcomputed tomographic (micro-CT) analysis of the lungs

**Figure 2:**
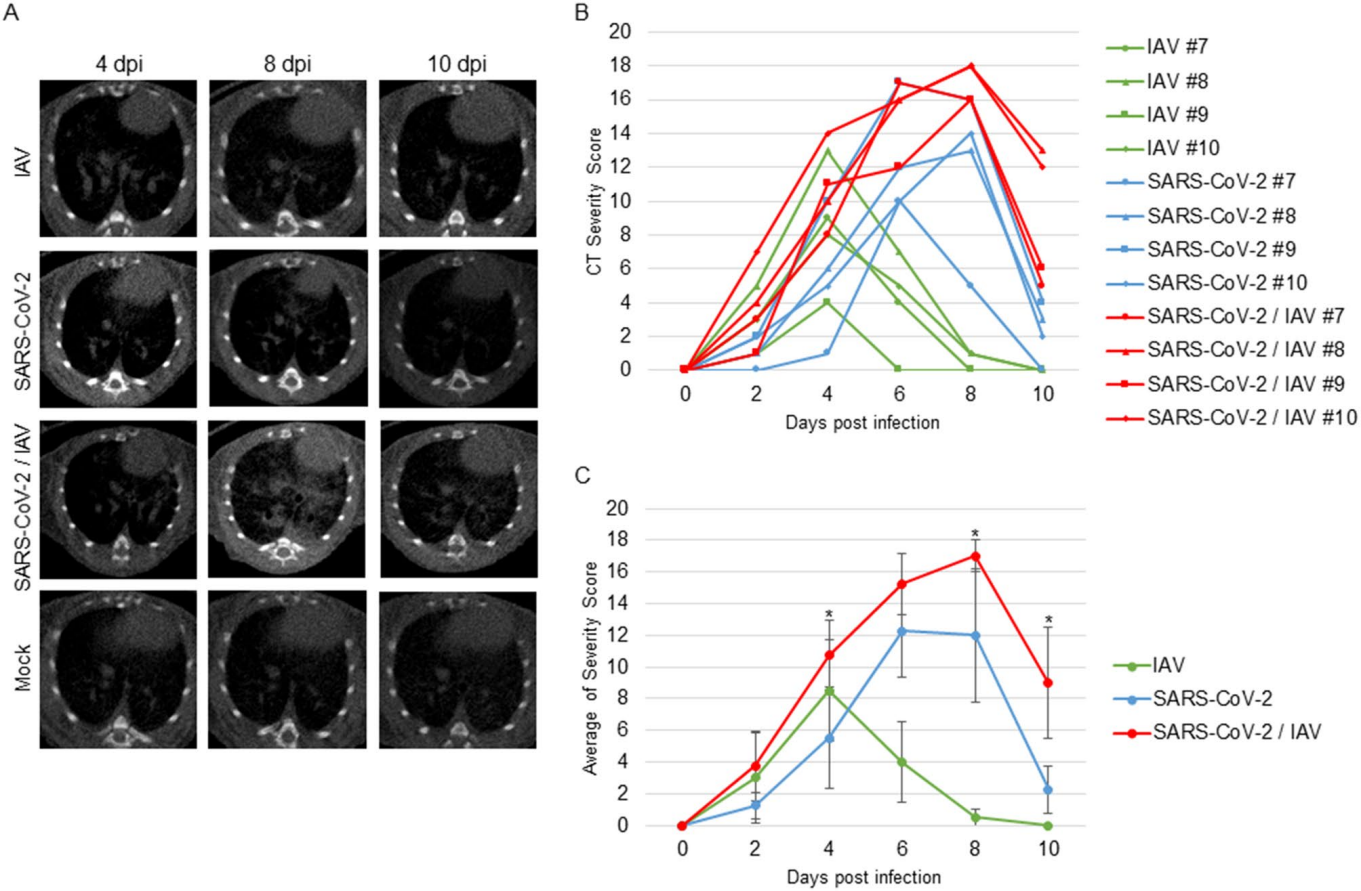
Assessment of pneumonia during infection using micro-CT. Lung abnormalities during infection were observed until 10 dpi using micro-CT. (**A**) CT images showed pneumonia caused by each virus at 4, 7, and 10 dpi. (**B**) CT severity score exhibited level of lung abnormalities. (**C**) Mean of CT severity score ± SD (n = 4 per group). Observation was performed every 2 days until 10 dpi. *P* values were calculated using Tukey’s multiple-comparison test (*P < 0.05; between SARS-CoV-2 and SARS-CoV-2/IAV).

To assess the influence of co-infection on lung pathology, we observed the abnormalities in the lungs during infection using in vivo micro-CT imaging. Hamsters of all three infected groups developed pneumonia (Fig. 2A). We evaluated the severity of pneumonia using the CT severity score^9,17^. At around 4 dpi, the hamsters infected with IAV exhibited the most severe lung abnormalities, while those in the SARS-CoV-2 single infection and co-infection groups showed the highest CT severity score at 6 and 8 dpi, respectively (Fig. 2B,C). Compared with SARS-CoV-2 single-infected hamsters, co-infected hamsters showed more severe pneumonia. Although both IAV and SARS-CoV-2 single-infected groups showed few lung abnormalities at 10 dpi, co-infected hamsters still showed pneumonia symptoms (Fig. 2C). These results indicated that co-infection with IAV and SARS-CoV-2 resulted in more severe and prolonged pneumonia compared with single virus infection.

### Histological analyses of respiratory organs

**Figure 3:**
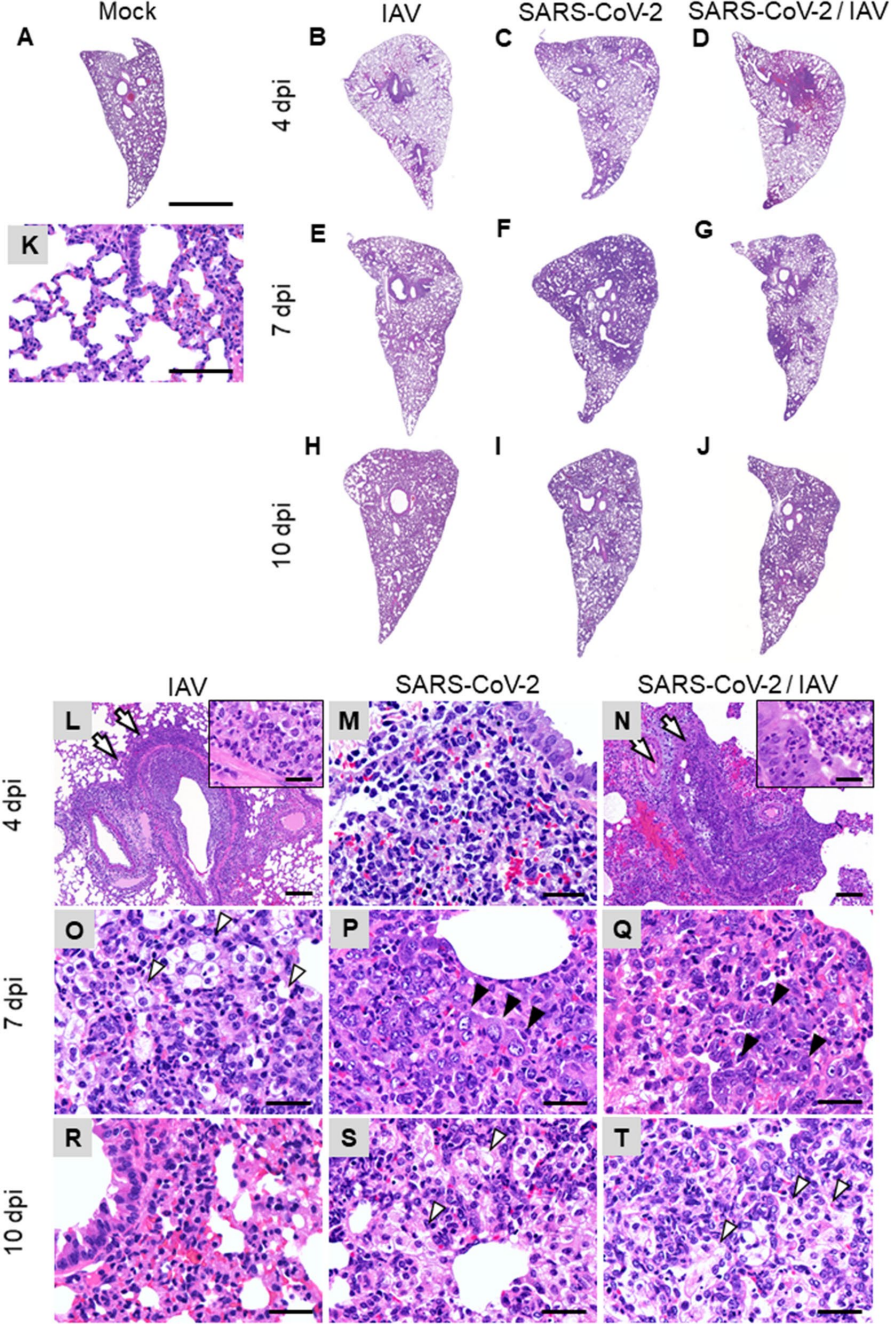
Histopathological analysis of lungs of infected hamsters. Lung sections were stained with hematoxylin and eosin (HE). Leftmost, second left, second right, and rightmost columns show representative sections of IAV, SARS-CoV-2, co-infected, and mock-infected hamsters, respectively. (**A**–**J**) Low-magnification images of the lung sections. (**K**–**T**) High-magnification images of (**A**–**J**). Arrows and inset in (**L**) and (**N**) indicate bronchointerstitial pneumonia (IAV pattern). White arrowheads in (**O**, **S**, and **T**) indicate alveolar macrophages. Black arrowheads in (**P** and **Q**) indicate type 2 pneumocytes. Scale bars: 4 mm (**A**), 200 μm (**K**), 100 μm (**L**, **N**), 50 μm (**M**, **O**–**T**), and 20 μm (inset in **L**, **N**).

**Figure 4:**
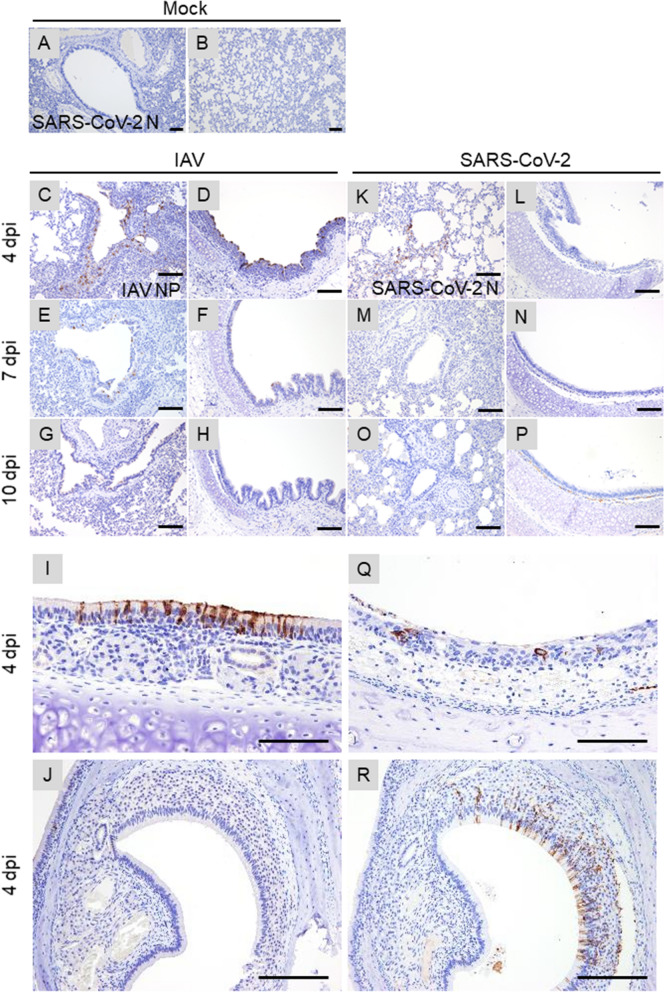
Immunohistochemical analysis of IAV NP and SARS N antigens in the tissue of IAV and SARS-CoV-2-infected hamsters, respectively. (**A**, **B**) Representative sections of tissue from the Mock-infected hamster stained with anti-SARS-CoV-2N antibody. (**C**–**J**) Representative sections of tissue from the IAV-infected hamster stained with anti-IAV NP antibody. (**K**–**R**) Representative sections of tissue from the SARS-CoV-2-infected hamster stained with anti-SARS-CoV-2N antibody. Lung are shown in (**A**–**C**, **E**, **G**, **K**, **M** and **O**). Trachea are shown in (**D**, **F**, **H**, **L**, **N** and **P**). Nasal mucosa are shown in (**I**, **Q**). Sections of the vomeronasal organ are shown in (**J** and **R**). Scale bars: 100 μm (**A**, **B**, **I**, **Q**) and 200 μm (**C**–**H**, **J**–**P**, **R**).

In the IAV single-infected group, hamsters showed acute bronchointerstitial pneumonia. At 4 dpi, mild, diffuse thickening of alveolar septa with infiltration of lymphocytes and macrophages were observed in the lungs (Fig. 3A,B,K,L). In some cases, focal bronchioles and alveoli were filled with degenerated neutrophils and cellular debris, then suppletive inflammation was observed in the surrounding area. Mild edema, hemorrhage, and fibrin exudation were occasionally observed. Moreover, at 7 dpi, foamy alveolar macrophages accumulated in the alveoli (Fig. 3E,O). At 10 dpi, the inflammatory lesion regressed and the alveolar septa were slightly thickened (Fig. 3H,R). In immunohistochemistry (IHC), tracheal epithelium, bronchial epithelium, and respiratory epithelial cells were positive for influenza A virus nucleoprotein (IAV NP) at 4, 7, and 10 dpi. In the foci of bronchointerstitial pneumonia, the affected bronchial epithelium was positive for IAV NP (“IAV pattern”). The number of NP-positive cells gradually decreased after 4 dpi (Fig. 4A–J). In the nasal mucosa, a few neutrophils and mononuclear cells infiltrated the submucosal layer. The nasal respiratory epithelium was occasionally positive for IAV NP at 4 dpi (Fig. 4I).

In the SARS-CoV-2 infected group, the hamsters showed severe interstitial pneumonia. At 4 dpi, multifocal thickening of alveolar septa, mild to moderate, with infiltration of lymphocytes and macrophages, was observed (Fig. 3C,M). The lesions were located around the terminal bronchioles. Mild edema and hemorrhage were presented in the parenchyma. At 7 dpi, the foci were coalescent, developing a diffuse interstitial pneumonia. Proliferation of type 2 pneumocytes in the alveoli and infiltration of neutrophils, lymphocytes, and macrophages were observed. The thickening of alveolar septa was so prominent that about 30–60% of alveolar space collapsed (“SARS-CoV-2 pattern”) (Fig. 3F,P). Bronchial epithelia also showed cell proliferation, and epithelial cells were swollen and arranged in multiple layers. At 10 dpi, type 2 pneumocytes were reduced, and mild inflammation and diffuse alveolar thickening were observed. In the alveolar space, foamy alveolar macrophages accumulated (Fig. 3I,S). In IHC, a few bronchial epithelial and respiratory epithelial cells were positive for SARS-CoV-2N at 4 dpi (Fig. 4K–P). In the nasal mucosa, mononuclear cells infiltrated the submucosal layer. In IHC, the supporting cells of the vomeronasal organ were strongly positive for SARS-CoV-2N at 4 dpi (Fig. 4P). The nasal respiratory epithelium was also positive, but comparatively rare (Fig. 4O).

**Figure 5:**
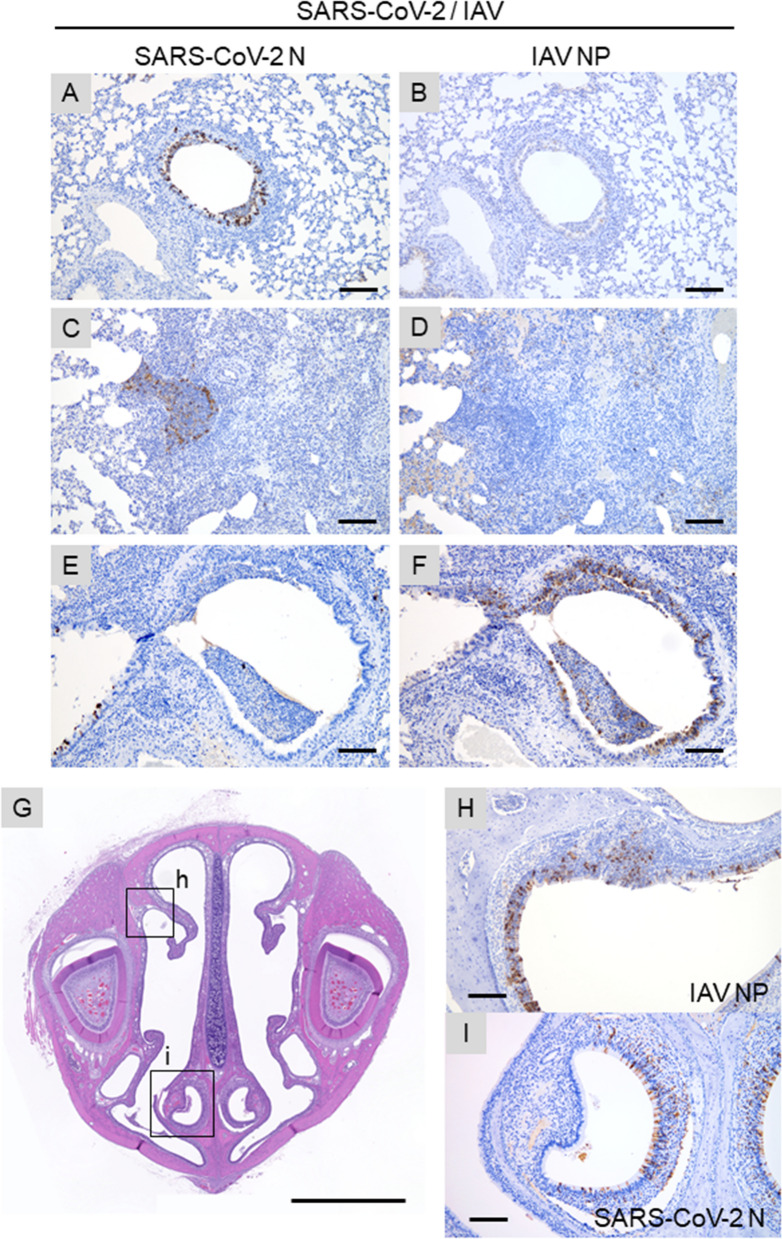
Immunohistochemical analysis of IAV NP and SARS N antigens in the tissue of co-infected hamsters. (**A**–**I**) Sections of the co-infected hamster at 4 dpi. (**A**–**F**) Representative serial sections of bronchi. (**G**) Representative coronal section of the head stained with HE. Nasal respiratory epithelium and vomeronasal organ are shown in (**H** and **I**), respectively. Sections in (**A**, **C**, **E**, and **H**) were stained with anti-IAV NP antibody. Sections in (**B**, **D**, **F**, and **I**) were stained with anti-SARS-CoV-2N antibody. Scale bars: 200 μm (**A**–**F**), 3 mm (**G**), and 100 μm (**H**, **I**).

In the lungs and other respiratory organs of co-infected hamsters, both SARS-CoV-2 and IAV infections were observed (Fig. 5). However, SARS-CoV-2 and IAV never co-existed at the same area in these organs. The examined lobes were clearly divided into SARS-CoV-2-patterned and IAV-patterned areas. At 4 dpi, focal inflammation with edema and hemorrhage was observed around the bronchioles (Fig. 3D,N). At 7 dpi, interstitial pneumonia with prominent proliferation of type 2 pneumocytes (SARS-CoV-2 pattern) and bronchointerstitial pneumonia (IAV pattern) were observed (Fig. 3G,Q). At 10 dpi, the alveolar septa were diffusely thickened with focal accumulation of foamy alveolar macrophages and atelectasis (Fig. 3J,T). As shown in Fig. 4, SARS-CoV-2 antigen was detected at 4 dpi, IAV antigen was detected at 4 and 7 dpi. At the surroundings of the SARS-CoV-2-positive bronchi, non-purulent interstitial pneumonia, as shown in SARS-CoV-2 single infection, was observed (Fig. 5A,B). By contrast, in the region surrounding the IAV-positive bronchi, acute bronchointerstitial pneumonia, as seen in IAV single infection, was observed (Fig. 5C,D). In the longitudinal section, bronchial epithelia were positive for both SARS-CoV-2 and IAV antigens, although each virus was independently distributed (not co-localized) (Fig. 5E,F). No viral antigen was detected at 10 dpi. In the nasal mucosa, SARS-CoV-2 and IAV were separately distributed in the nasal respiratory epithelium without co-localization at 4 dpi (Fig. 5G–I). SARS-CoV-2 was also observed in the supporting cells of vomeronasal organ (Fig. 5I).

There was no considerable difference in the pathology and virus replication sites in other examined organs in the single and co-infection group (data not shown).

### Induction of cytokines and chemokines

To examine whether co-infection alters the induction patterns of cytokines and chemokines, we measured the serum levels of IP-10, MCP-1, TNF-α, IL-1-β, IL-6, IL-10, and IFN-γ over the course of infection using ELISA.

**Figure 6:**
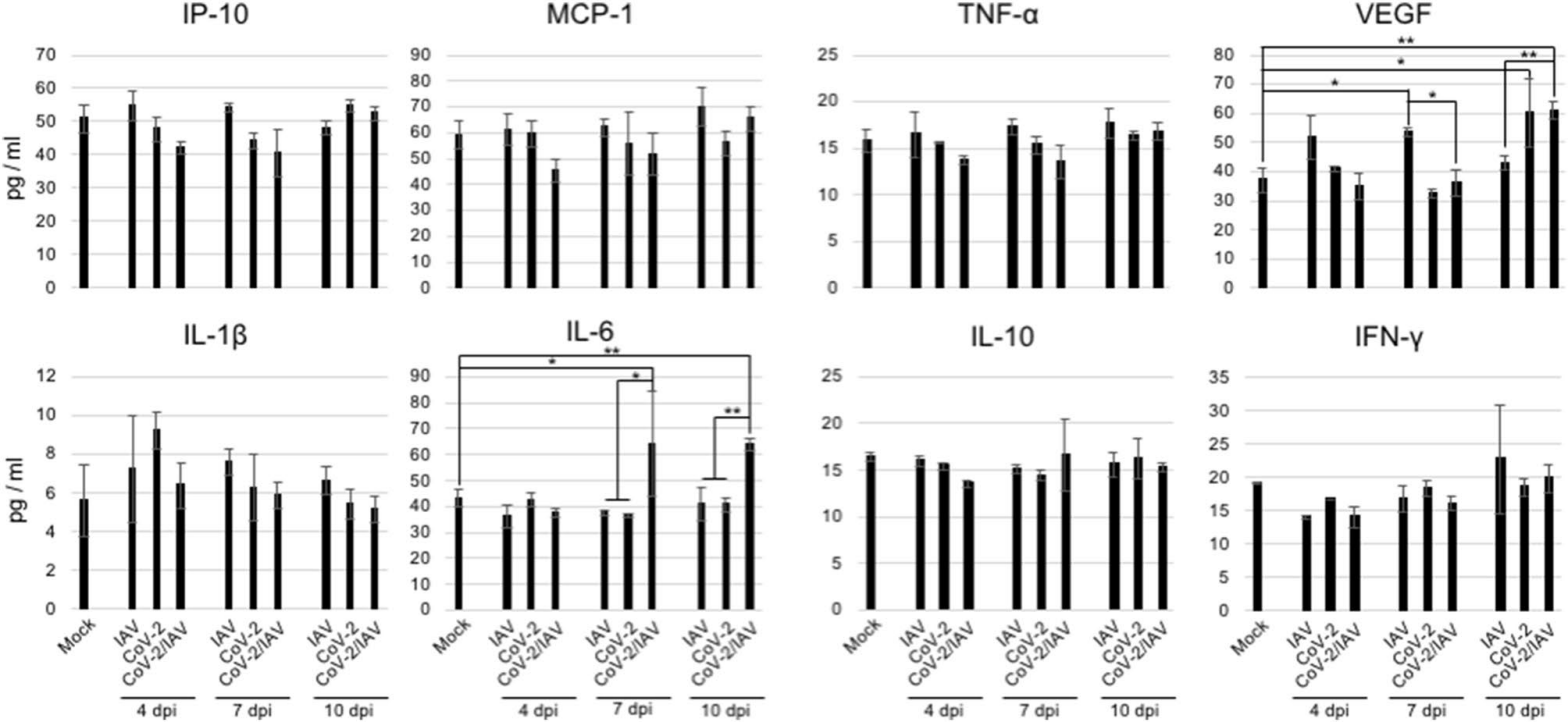
Measurement of inflammatory and anti-inflammatory cytokines and chemokines in the serum. Inflammatory and anti-inflammatory cytokines and chemokines were quantified by ELISA. Data are presented as the mean value ± SD (n = 3 per group). *P* values were calculated using Tukey’s multiple-comparison test (*P < 0.05; **P < 0.01).

IL-6, an inflammatory cytokine, was significantly elevated at 7 and 10 dpi in the co-infection group, but not in the other groups (Fig. 6). The abundance of IL-10, an anti-inflammatory cytokine, showed no apparent difference among the three groups. We also found that the serum levels of vascular endothelial growth factor (VEGF) were increased at 4 and 7 dpi in the IAV group and at 10 dpi in the SARS-CoV-2 and co-infection groups.

## Discussion

In this study, we showed that co-infection of IAV in SARS-CoV-2-infected hamsters causes more severe body weight loss, delayed recovery, and more severe and prolonged pneumonia than those observed with single infection by SARS-CoV-2 (Table 1). This finding suggests a correlation between body weight loss and the severity of pneumonia determined by micro-CT imaging. Consistent with these enhancements of pathology, co-infected hamsters showed pathological features of both IAV and SARS-CoV-2 infections and high levels of IL-6.

**Table 1:** Summary of disease markers.

	Body weight loss	Viral titers (log_10_)				CT score	Histopathological findings	Cytokine levels
		IAV		SARS-CoV-2				
		4 dpi	7 dpi	4 dpi	7 dpi			
IAV	− 3% (3 dpi)	6.9	2.7	–	–	8.5 (4 dpi)	Bronchointerstitial pneumonia	↗ VEGF (4,7 dpi)
SARS-CoV-2	− 10% (6 dpi)	–	–	11	7.1	12 (6 dpi)	Interstitial pneumonia with prominent proliferation of type 2 pneumocytes	↗ VEGF(10 dpi)
Co-infection	− 14% (7 dpi)	7.0	3.7	11	7.3	17 (8 dpi)	Both IAV and SARS-CoV-2 pathology	↗ VEGF(10 dpi) ↗ IL-6 (7,10 dpi)

Hamsters were intranasally inoculated with 1 × 10^5^ PFU of IAV (PR8), 3 × 10^5^ PFU of SARS-CoV-2, or a mixture of both viruses (co-infection). The peak percentage of body weight loss and days post infection (dpi) are shown under “Body weight loss”. The numbers in viral loads indicate the gene copies per lung weight (mg). The peak CT severity score and dpi are displayed under “CT score”. The cytokines that showed significantly increased levels after the infection and dpi are shown under “Cytokine levels”.

As previously reported, hamsters were susceptible to both IAV and SARS-CoV-2 infections and both viruses could efficiently replicate in the lung^8,9,16^. The virus titers of IAV and SARS-CoV-2 in the lungs of infected hamsters were the highest at 3 dpi and declined at 6 dpi. Our results also showed similar growth patterns of both viruses in the lung (Fig. 1B,C). These results indicate that both viruses rapidly spread to the lung and efficiently replicate there.

The IAV and SARS-CoV-2 loads in the lungs of co-infected hamsters were similar to the viral load in hamsters single-infected with each virus (Fig. 1B,C), indicating that both viruses can efficiently spread in the lungs without the mutual interference that is often observed in virus co-infection^7^. Viral interference was not observed in this study, at either the individual or organ level. Notably, IAV and SARS-CoV-2 were not detected at the same sites in the respiratory organs of co-infected hamsters (Figs. 4 and 5), suggesting either that these viruses have different in vivo cell tropism or that each virus inhibits the infection and/or replication of the other within a cell or adjacent area in the organ. A previous report showed that IAV significantly downregulated the expression of ACE2, a cellular receptor of SARS-CoV-2, via the proteasome pathway in vitro^18^. Therefore, the downregulation of this receptor by IAV may prevent the growth of SARS-CoV-2 and the co-existence of SARS-CoV-2 and IAV in the same area in organs in vivo.

Significant increase in IL-6 was detected in the sera of the hamsters co-infected with SARS-CoV-2 and IAV at 7 and 10 dpi (Fig. 6, Table 1). IL-6 signaling play crucial roles in vascular endothelial cell dysfunction in cytokine release syndrome (CRS) and in the progression of acute inflammatory diseases such as sepsis and acute respiratory distress syndrome^19^. Therefore, the increase in serum IL-6 may be involved in the increased severity of pneumonia observed in this study. Moreover, serum IL-6 level is a known predictive biomarker of COVID-19 severity^20^. Collectively, these findings are consistent with the increased disease severity in co-infected hamsters, compared with that in single-infected hamsters, observed in the present study.

In this study, VEGF significantly increased in hamster sera at 4 and 7 dpi in IAV-infected hamsters and at 10 dpi in SARS-CoV-2- and co-infected hamsters, respectively (Fig. 6, Table 1). In IAV-infected hamsters, pneumonia severity peaked at 4 dpi, whereas in the SARS-CoV-2 and co-infection groups, pneumonia severity peaked at 6 and 8 dpi, respectively (Fig. 2). VEGF production appears to be induced in order to recover from the damage caused by infection.

Viral infection of vomeronasal cells is generally rare, although herpes simplex virus has been reported to replicate in these cells^21^. As shown in Fig. 5G and I, SARS-CoV-2, but not IAV, was detected in the supporting cells of the vomeronasal organ. This finding may provide a clue for understanding the occurrence of dysosmia in COVID-19 patients.

Our data showed that hamsters infected with the IAV PR8 strain exhibited body weight loss at an early phase of infection and interstitial pneumonia as observed in human infection. In addition, we examined if the results from hamsters co-infected with a mouse-adapted IAV PR8 strain were similar to those from hamsters co-infected with the influenza A (H1N1) pdm09 strain, which has been recently known to cause human infections. Unlike a mouse-adapted PR8 strain, pdm09 strain did not cause body weight loss and any respiratory symptom (data not shown). Previous report also showed that, in the hamsters infected with pdm09 strains, the virus antigens were detected mainly in the olfactory epithelia rather than in the respiratory epithelia, and any respiratory symptom was not observed^16^, suggesting that the hamster infected with mouse-adapted PR8 strain, but not pdm09 strain, should be appropriate as a pathological model of influenza.

The hamsters co-infected with SARS-CoV-2 and IAV showed more severe and prolonged pneumonia. This observation is also consistent by a case report of a co-infected patient (32-year-old man) who exhibited severe pneumonia and died of persistent respiratory failure^22^. Clinical report also showed that COVID-19 patients with influenza exhibited more severe inflammation and organ injury^23^. However, another report suggested that co-infection with influenza was not associated with increased disease severity of COVID-19 pneumonia^24^. Therefore, further studies might be needed to understand effects of co-infection on patients.

Taken together, our data strongly suggest that co-infection with IAV poses a serious risk to SARS-CoV-2 infected patients and should be taken into consideration in the treatment and management of these patients.

## Methods

### Cells and viruses

VeroE6/TMPRSS2 cells, a VeroE6 cell line constitutively expressing TMPRSS2 provided by National Institutes of Biomedical Innovation, Health and Nutrition (NIBIOHN), Osaka, Japan^25^, and Madin-Darby canine kidney (MDCK) cells, kindly provided by Yoshihiro Kawaoka, University of Tokyo, Tokyo, Japan, were cultured in Dulbecco’s modified Eagle medium (DMEM; Sigma Aldrich, St. Louis, MO, USA) supplemented with 10% fetal calf serum (FCS) and minimal essential medium (MEM; Sigma Aldrich) with 5% FCS, respectively. SARS-CoV-2 strain, JPN/NGS/SC-1/2020 (GISAID Accession ID: EPI_ISL_481254), was isolated from a COVID-19 patient and propagated in Vero/TMPRSS2 cells in DMEM containing 1% FCS. Mouse-adapted influenza A/Puerto Rico/8/34 (H1N1) virus was propagated in MDCK cells in MEM containing 1 × Vitamin (Gibco, Thermo Fisher Scientific, Waltham, MA, USA), 0.1% BSA (Sigma Aldrich), 1 × MEM Non-Essential Amino Acids (NEAA; Gibco) and 0.00075% Trypsin (Sigma Aldrich).

### Infection of Syrian hamsters

Five-week-old female Syrian hamsters (Japan SLC Inc., Shizuoka, Japan) were used in this study. In infection experiments, hamsters were intranasally inoculated with 3 × 10^5^ PFU (in 100 µl) of SARS-CoV-2 and/or 1 × 10^5^ PFU (in 100 µl) of IAV. Body weight was monitored daily for 10 days. At 4, 7, or 10 dpi, hamsters were euthanized by exsanguination under deep isoflurane anesthetize, and serum and organs were collected. For pathological examinations, organs were fixed with a 10% formalin neutral buffer solution (FUJIFILM Wako Pure Chemical, Tokyo, Japan). For quantification of the viral RNA in organs, the collected organs were immersed in lysis buffer (Thermo Fisher Scientific) and then disrupted by high-speed shaking using a TissueLyser II (Qiagen, Hilden, Germany). All experiments with hamsters were performed in accordance with the ARRIVE guidelines.

### Quantitative real-time RT-PCR (RT-qPCR) of viral RNA

Total RNA was extracted from organs by Pure link RNA kit (Thermo Fisher Scientific). RT-qPCR was performed with viral gene specific primers^26,27^ using the One Step PrimeScript™ III RT-qPCR Mix (Takara Bio Inc., Shiga, Japan) according to the manufacturer’s instructions. The N gene of SARS-CoV-2 or the M gene of IAV was amplified using the following paired primers and probes; forward primer 5′-AAATTTTGGGGACCAGGAAC-3′, reverse primer 5′-TGGCAGCTGTGTAGGTCAAC-3′, probe 5′-(FAM)ATGTCGCGCATTGGCATGGA(BHQ)-3′ (SARS-CoV-2) or forward primer 5′-CCMAGGTCGAAACGTAYGTTCTCTCTATC-3′, reverse primer 5′-TGACAGRATYGGTCTTGTCTTTAGCCAYTCCA-3′, probe 5′-(FAM)ATYTCGGCTTTGAGGGGGCCTG(MGB)-3′ (IAV), respectively. The following template cDNA were used for standard; 5′-GCCAGTGAATTGTAATACGACTCACTATAGGGCGAAGGAAATTTTGGGGACCAGGAACTAATCAGACAAGGAACTGATTACAAACATTGGCCGCAAATTGCACAATTTGCCCCCAGCGCTTCAGCGTTCTTCGGAATGTCGCGCATTGGCATGGAAGTCACACCTTCGGGAACGTGGTTGACCTACACAGGTGCCATCAA-3′ (SARS-CoV-2) and 5′-AGTCTTCTAACCGAGGTCGAAACGTACGTTCTCTCTATCATCCCGTCAGGCCCCCTCAAAGCCGAGATCGCACAGAGACTTGAAGATGTCTTTGCAGGGAAGAACACCGATCTTGAGGTTCTCATGGAATGGCTAAAGACAAGACCAATCCTGTCACCTCTGACTA-3′ (IAV).

### micro-CT imaging

CT images were acquired using Triumph combined PET/SPECT/CT systems (TriFoil Imaging, Chatsworth, CA, USA). The animals were anesthetized with 1.5% isoflurane, and CT was performed for observation of lung field. Acquired CT data were processed using OsiriX MD (Pixmeo, Bernex, Switzerland). The severity of pneumonia was determined as a CT severity score according to previous methods^9,17^.

### Histopathological analysis

Organs (liver, spleen, kidney, heart, lung, pancreas, duodenum, ileum, colon, brain, and nasal part of frontal bone) were fixed in neutral buffered formalin and processed using routine methods. After fixation, the frontal bone was decalcified with 10% EDTA solution (pH 7.4). Serial sections, 4 μm thick, were sliced from paraffin-embedded tissues and stained with hematoxylin and eosin (HE). IHC was performed using mouse anti-SARS-CoV-2N monoclonal antibody (Thermo Fisher Scientific) or rabbit anti-influenza A virus NP polyclonal antibody (Genetex Inc, Irvine, CA, USA). Antigen retrieval was performed by microwave heating with citrate buffer (pH 6.0) at 95 °C for 15 min. Signals were visualized using the Envision system (Agilent Technologies, Santa Clara, CA, USA) and 3,3’-diaminobenzidine.

### ELISA for quantitation of cytokines and chemokines

Blood from hamsters was centrifuged (1000×*g*, 15 min, RT) and serum was collected. Cytokines and chemokines were quantified using the Hamster ELISA kit (MyBioSource, Inc., San Diego, CA, USA) according to the manufacturer’s instructions.

### Statistical analysis

Statistical evaluation of the differences between the groups was performed using the Student’s t-test and the Tukey’s multiple-comparison test by using R software version 3.2.2. *P* < 0.05 was considered significant.

### Ethics statement

Our protocol for the experiments involving hamsters followed the Nagasaki University Regulations for Animal Care and Use and were approved by the Animal Experiment Committee of Nagasaki University (approval number 2003031600-4). All experiments with SARS-CoV-2 were performed in BSL-3 laboratories at Nagasaki University.
